# The mechanism of peach kernel and safflower herb-pair for the treatment of liver fibrosis based on network pharmacology and molecular docking technology: A review

**DOI:** 10.1097/MD.0000000000033593

**Published:** 2023-04-21

**Authors:** Long Huang, Qingsheng Yu, Hui Peng, Zhou Zhen

**Affiliations:** a Department of No.1 Surgery, The First Hospital Affiliated to Anhui University of Traditional Chinese Medicine, Hefei, Anhui Province, China; b Department of Surgery, The Second Hospital Affiliated to Anhui University of Traditional Chinese Medicine, Hefei, Anhui Province, China.

**Keywords:** inflammation, liver fibrosis, molecular docking, network pharmacology, peach kernel, safflower

## Abstract

Peach kernel and safflower herb-pair (PKSH) are widely used in traditional Chinese medicine for the treatment of liver fibrosis. Therefore, network pharmacology was performed to explore potential therapeutic targets and pharmacological mechanisms of PKSH. The active components of PKSH from the Traditional Chinese Medicine Systems Pharmacology Database and Analysis Platform database and potential targets of liver fibrosis from the Online Mendelian Inheritance in Man, Pharmacogenetics and Pharmacogenomics Knowledge Base, GeneCards, and DrugBank Database were identified. The protein-protein interaction network was constructed using Cytoscape (v3.8.0). Gene Ontology and Kyoto Encyclopedia of Genes and Genomes pathway enrichment analyses were performed for the treatment of liver fibrosis, and molecular docking was carried out to verify the results of network pharmacology analysis. After screening disease-related genes, 179 intersection genes overlapped between 196 target proteins of the active compound and 9189 potential disease targets. Furthermore, we obtained 15 hub nodes and 146 edges to establish a related network diagram using CytoNCA. 2559 Gene Ontology biological processes underlying PKSH have been explored for the treatment of liver fibrosis, in which the response to oxidative stress plays a vital role. Furthermore, Kyoto Encyclopedia of Genes and Genomes enrichment analysis revealed that PKSH might play a role in inhibiting liver fibrosis, mainly through the PI3K-Akt signaling pathway. PKSH can regulate the response to oxidative stress through the PI3K-Akt signaling pathway for the treatment of liver fibrosis. The main bioactive components in PKSH, including quercetin and luteolin, can activate the PI3K-Akt signaling pathway by binding with the hub targets of the disease, which may provide insights into drug development for liver fibrosis.

## 1. Introduction

Liver fibrosis, the main pathological change in the development of chronic liver disease, manifests as excessive proliferation and deposition of extracellular matrix (ECM) in liver tissue. The progression of liver fibrosis can lead to abnormal changes in the structure of the liver tissue and affect the normal physiological function of the liver. Furthermore, the liver parenchyma is gradually replaced by scar tissue formed by the ECM, which eventually leads to liver cirrhosis and even cancer.^[1]^

Most chronic liver diseases are accompanied by liver fibrosis owing to the long-term stimulation of pathogenic factors.^[2]^ Long-term stimulation by various pathogenic factors, including hepatitis virus, ethanol, toxins, parasites, and autoimmune abnormalities, can lead to the progression of liver fibrosis.

Chronic liver injury can lead to liver cirrhosis, which seriously affects the health and quality of life of patients. Of approximately 6 billion people in the world, about 2 billion have evidence of hepatitis B virus (HBV) infection, of which 350 to 400 million have chronic HBV infections, accounting for approximately 6% of the global population. Prospective studies have shown that the annual incidence of chronic HBV infection developing into cirrhosis is 2 to 10%, and liver cirrhosis is a common cause of death in gastrointestinal diseases.^[3]^

Research shows that etiological treatment can help inhibit or even reverse liver fibrosis, which can effectively inhibit viral replication and reverse liver fibrosis in chronic hepatitis B.^[4]^ However, there are still some limitations in the treatment of liver fibrosis. It is important to treat liver fibrosis in patients with chronic liver disease by inhibiting the proliferation of fibrotic tissue and promoting the degradation of ECM.^[5]^ Liver cirrhosis is the outcome of the development of liver fibrosis, and the prevention or reversal of liver fibrosis is an important treatment.

Owing to the complex pathological mechanism of liver fibrosis, drugs developed for a single target are difficult to work with in clinical practice; therefore, there is still no biological drug with definite efficacy available to inhibit the proliferation of fibrotic tissue and promote the degradation of ECM. Recent decades of research and applications have shown that Chinese medicine is effective in the treatment of chronic liver disease, especially in the prevention and treatment of liver fibrosis.^[6]^ Traditional Chinese medicine (TCM) against liver fibrosis has been widely used in clinical practice for >20 years, and a large amount of clinical data has been accumulated. Therefore, Chinese medicine has attracted increasing attention because it provides more opportunities for the treatment of chronic liver diseases.

PI3K-Akt signaling pathway is involved in inflammation.^[7]^ Furthermore, inflammation is also involved in diseases that may cause liver fibrosis, such as hepatosteatosis, and viral and autoimmune hepatitis.^[8–10]^ Thus, studying the effects of peach kernel and safflower herb-pair (PKSH) on inhibiting inflammatory response through the PI3K-AKT pathway for the treatment of liver fibrosis makes sense. The formula composed of PKSH is widely used in China to treat chronic hepatitis and liver fibrosis and has a satisfactory therapeutic effect.^[11,12]^ PKSH improves liver fibrosis by promoting blood circulation.^[13]^ However, few studies have addressed the association between cirrhosis and PKSH. The mechanism by which PKSH plays an antifibrotic role remains to be elucidated. Therefore, it is important to clarify the specific mechanisms of PKSH in the treatment of liver fibrosis by using network pharmacology.

Network pharmacology systematically predicts possible mechanisms and discusses the relationship between medicine and diseases by constructing a “drug-gene-target-disease” network, which conforms to the holistic view of TCM theory.^[14]^ Molecular docking is a theoretical method used to study the interaction and recognition between protein receptors and small molecule ligands, which can predict the binding mode and strength of drug ligands and targets. In general, network pharmacology and molecular docking in combination can be used to explore the potential mechanism of action of active compounds in TCM and to verify target binding.

This study aimed to explore the key components of PKSH through network pharmacology and investigate the key targets and possible mechanisms of action in the treatment of liver fibrosis. In this study, we conducted a comprehensive network pharmacology study to investigate the possible pharmacological mechanism of PKSH in treating liver fibrosis based on Network Pharmacology and Molecular Docking Technology and comprehensively and systematically characterized the intervention and impact of drugs on diseases.

## 2. Methods

### 2.1. Screening of compound components from peach kernel and safflower

The active components and targets of PKSH were obtained from the Traditional Chinese Medicine Systems Pharmacology Database and Analysis Platform (TCMSP) (http://tcmspw.com/tcmsp.php). The various compounds in PKSH were screened according to pharmacokinetic absorption (A), distribution (D), metabolism (M), and excretion (E) parameters, so molecules with oral bioavailability ≥ 30% and drug-likeness ≥ 0.18 were defined as active molecules. The corresponding target gene information was searched for using the UniProt database (https://www.uniprot.org/). The active compound targets in PKSH were screened in TCMSP^[15]^ and annotated using UniPort^[16]^ and GeneCards databases.^[17]^

### 2.2. Acquisition of disease targets

The gene targets of liver fibrosis were collected from 4 databases by querying with the keywords “liver fibrosis”: Online Mendelian Inheritance in Man database (OMIM, https://omim.org/); GeneCards Human Gene database (GeneCards, https://www.genecards.org/); DrugBank Database (https://go.drugbank.com/); and Pharmacogenetics and Pharmacogenomics Knowledge Base (https://www.pharmgkb.org/). Furthermore, we removed duplicate targets and merged all collected targets from the 4 databases. Finally, the gene targets were standardized using the UniProt database, and the species was selected as “Homo sapiens.”^[18]^

### 2.3. Construction of the network diagram

The overlapping intersecting genes between the active compound targets of PKSH and target genes in liver fibrosis were elucidated. Based on the compounds and targets of PKSH in the treatment of liver fibrosis, a drug-compound-gene target network was constructed and visualized using Cytoscape 3.8.0 (National Institute of General Medical Sciences (NIGMS), USA).^[19]^ Target molecules, including compounds, proteins, and disease genes, were represented as nodes. The interactions between the nodes are represented as edges. Based on this network, we systematically analyzed the relationship between components, targets, and diseases using a drug-compounds-gene targets network diagram.

#### 2.3.1. Protein-protein interaction network of target protein interaction.

The protein-protein interaction (PPI) network construction of target proteins in treating liver fibrosis was conducted using Online STRING 11.0 (https://string-db.org/). The liver fibrosis-related target genes of PKSH were input into the STRING database for amplification and prediction, and the interaction of target proteins in the network diagram was obtained. The confidence score in PPIs was set to 0.9 for the highest confidence from the STRING database.^[20]^ The amplified target proteins of PKSH for the treatment of liver fibrosis in the STRING database were input into Cytoscape software V3.8.0, and the protein interaction network was obtained.

#### 2.3.2. Screening of core clusters and key targets.

Based on the 6 parameters of “Betweenness,” “Closeness,” ”Degree,” “Eigenvector,” ”Local Average Connectivity-based method,” and “Network” by CytoNCA tool, we conduct topology analysis on the PPI network to obtain the hub nodes.^[21]^ The median values of these parameters were calculated, and all nodes whose 6 parameters were greater than the median values were selected as hub nodes.

### 2.4. Gene Ontology and Kyoto Encyclopedia of Genes and Genomes pathway enrichment analyses

Gene Ontology (GO) functional annotation and Kyoto Encyclopedia of Genes and Genomes (KEGG) pathway analysis were performed using the cluster Profiler R package 4.1.2 (Bell Laboratories, USA), and the enrichment analysis results were visualized.^[22]^

### 2.5. Molecular docking verification

The structures of all compounds were downloaded from the PubChem database (https://pubchem.ncbi.nlm.nih.gov/), and the structures of key targets were downloaded from the Protein Database database (http://www.rcsb.org/pdb/home/home.do). The Ligand Docking module was used to verify the reliability of the results using AutoDockTools Version 1.5.6 (Molecular Graphics Laboratory, The Scripps Research Institute, USA), and the bonding activity of the compound to the key targets was evaluated using the docking score.^[23]^ The higher the absolute value of the docking score, the stronger the binding ability of the small molecules to protease targets.

## 3. Results

### 3.1. The predicted targets of the PKSH

The TCMSP 2.3 was used to determine the chemical composition of the drug and its targets. We screened various compounds in PKSH according to pharmacokinetic absorption (A), distribution (D), metabolism (M), and excretion (E) parameters. Based on the TCMSP database, there were 23 and 22 compounds in PKSH respectively, which met the criteria of oral bioavailability ≥ 30% and drug-likeness ≥ 0.18. The top 33 compounds of degree in PKSH are listed in Table 1. Among the 44 active compounds, we identified 196 potential related targets, including 193 in safflower and 40 in peach kernel.

**Table 1:** Potential effective compounds of peach kernel and safflower herb-pair.

Mol ID	Molecule name	Herb	OB (%)	DL	Degree
MOL000098	Quercetin	Honghua	46.43	0.3	130
MOL000006	Luteolin	Honghua	36.16	0.3	51
MOL000422	Kaempferol	Honghua	41.88	0.2	47
MOL002714	Baicalein	Honghua	33.52	0.2	29
MOL000449	Stigmasterol	Honghua	43.83	0.8	25
MOL000358	Beta-sitosterol	Honghua/Taoren	36.91	0.8	25
MOL002773	Beta-carotene	Honghua	37.18	0.6	20
MOL000296	Hederagenin	Taoren	36.91	0.8	13
MOL002712	6-Hydroxykaempferol	Honghua	62.13	0.3	8
MOL001358	Gibberellin 7	Taoren	73.8	0.5	6
MOL002721	Quercetagetin	Honghua	45.01	0.3	6
MOL001340	GA120	Taoren	84.85	0.5	5
MOL001328	2,3-Didehydro GA70	Taoren	63.29	0.5	5
MOL001323	Sitosterol alpha1	Taoren	43.28	0.8	5
MOL000493	Campesterol	Taoren	37.58	0.7	4
MOL002757	7,8-Dimethyl-1H-pyrimido[5,6-g]quinoxaline-2,4-dione	Honghua	45.75	0.2	4
MOL001368	3-o-p-Coumaroylquinic acid	Taoren	37.63	0.3	3
MOL002717	qt_carthamone	Honghua	51.03	0.2	3
MOL001329	2,3-Didehydro GA77	Taoren	88.08	0.5	3
MOL002695	Lignan	Honghua	43.32	0.7	3
MOL001361	GA87	Taoren	68.85	0.6	2
MOL001355	GA63	Taoren	65.54	0.5	2
MOL000953	CLR	Honghua	37.87	0.7	2
MOL001353	GA60	Taoren	93.17	0.5	2
MOL001352	GA54	Taoren	64.21	0.5	2
MOL001351	Gibberellin A44	Taoren	101.61	0.5	2
MOL001349	4a-formyl-7alpha-hydroxy-1-methyl-8-methylidene-4aalpha,4bbeta-gibbane-1alpha,10beta-dicarboxylic acid	Taoren	88.6	0.5	2
MOL001360	GA77	Taoren	87.89	0.5	1
MOL001344	GA122-isolactone	Taoren	88.11	0.5	1
MOL002710	Pyrethrin II	Honghua	48.36	0.4	1
MOL001342	GA121-isolactone	Taoren	72.7	0.5	1
MOL002694	4-[(e)-4-(3,5-dimethoxy-4-oxo-1-cyclohexa-2,5-dienylidene)but-2-enylidene]-2,6-dimethoxycyclohexa-2,5-dien-1-one	Honghua	48.47	0.4	1
MOL001771	Poriferast-5-en-3β-ol	Honghua	36.91	0.8	1

DL = drug-likeness, OB = oral bioavailability.

### 3.2. Screening of targets related to liver fibrosis

**Figure 1.**
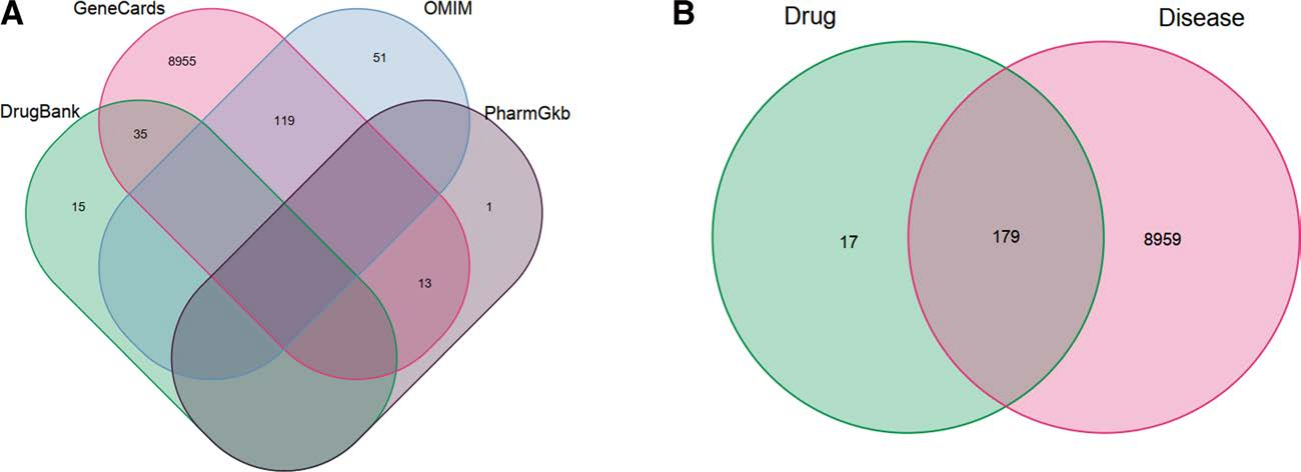
Overlapped genes between targets of active compounds in peach kernel and safflower herb-pair and liver fibrosis-related genes from the GeneCards, DrugBank, OMIM, and PharmGkb databases. (A) Liver fibrosis-related genes. (B) overlapped genes. PharmGkb = Pharmacogenetics and Pharmacogenomics Knowledge Base.

We screened disease-related genes from the GeneCards, DrugBank, OMIM, and Pharmacogenetics and Pharmacogenomics Knowledge Base databases, there were 179 intersection genes overlapped between 196 target proteins of the active compound and 9189 potential disease targets (Fig. 1). The main overlapping genes between the target genes of active compounds and liver fibrosis-related genes included PTGS2, GABRA1, PTGS1, HSP90AB1, PGR, CHRM1, PRSS1, CHRM2, CASP3, and DPP4 (Table 2).

**Table 2:** Information on 30 core targets.

Name	Description	UniProt	Length	Degree
PTGS2	Prostaglandin G/H synthase 2	P35354	604	25
GABRA1	Gamma-aminobutyric acid receptor subunit alpha-1	P14867	456	14
PTGS1	Prostaglandin G/H synthase 1	P23219	599	13
HSP90AB1	Heat shock protein HSP 90-beta	P08238	724	12
PGR	Progesterone receptor	P06401	933	11
CHRM1	Muscarinic acetylcholine receptor M1	P11229	460	7
PRSS1	Alpha-1-antitrypsin	P01009	418	7
CHRM2	Muscarinic acetylcholine receptor M2	P08172	466	6
CASP3	Caspase-3	P42574	277	6
DPP4	Dipeptidyl peptidase 4	P27487	766	6
AR	Androgen receptor	P10275	920	6
NR3C2	Mineralocorticoid receptor	P08235	984	5
CHRM3	Muscarinic acetylcholine receptor M3	P20309	590	5
JUN	Transcription factor AP-1	P05412	331	5
BCL2	Apoptosis regulator Bcl-2	P10415	239	5
AKT1	RAC-alpha serine/threonine-protein kinase	P31749	480	5
PPARG	Peroxisome proliferator-activated receptor gamma	P37231	505	5
ADRB2	β-2 adrenergic receptor	P07550	413	4
ADRA1B	Alpha-1B adrenergic receptor	P35368	520	4
SCN5A	Sodium channel protein type 5 subunit alpha	Q14524	2016	4
HMOX1	Heme oxygenase 1	P09601	288	4
MMP1	Interstitial collagenase	P03956	469	4
CASP9	Caspase-9	P55211	416	4
BAX	Apoptosis regulator BAX	Q07812	192	4
VEGFA	Vascular endothelial growth factor A	P15692	232	4
RELA	RelA-associated inhibitor	Q8WUF5	828	4
RXRA	Retinoic acid receptor RXR-alpha	P19793	462	3
ADH1C	Alcohol dehydrogenase 1C	P00326	375	3
SLC2A4	Solute carrier family 2, facilitated glucose transporter member 4	Q9NR83	387	3
GSTP1	Glutathione s-transferase P	P09211	210	3

### 3.3. The construction of the network diagram

**Figure 2.**
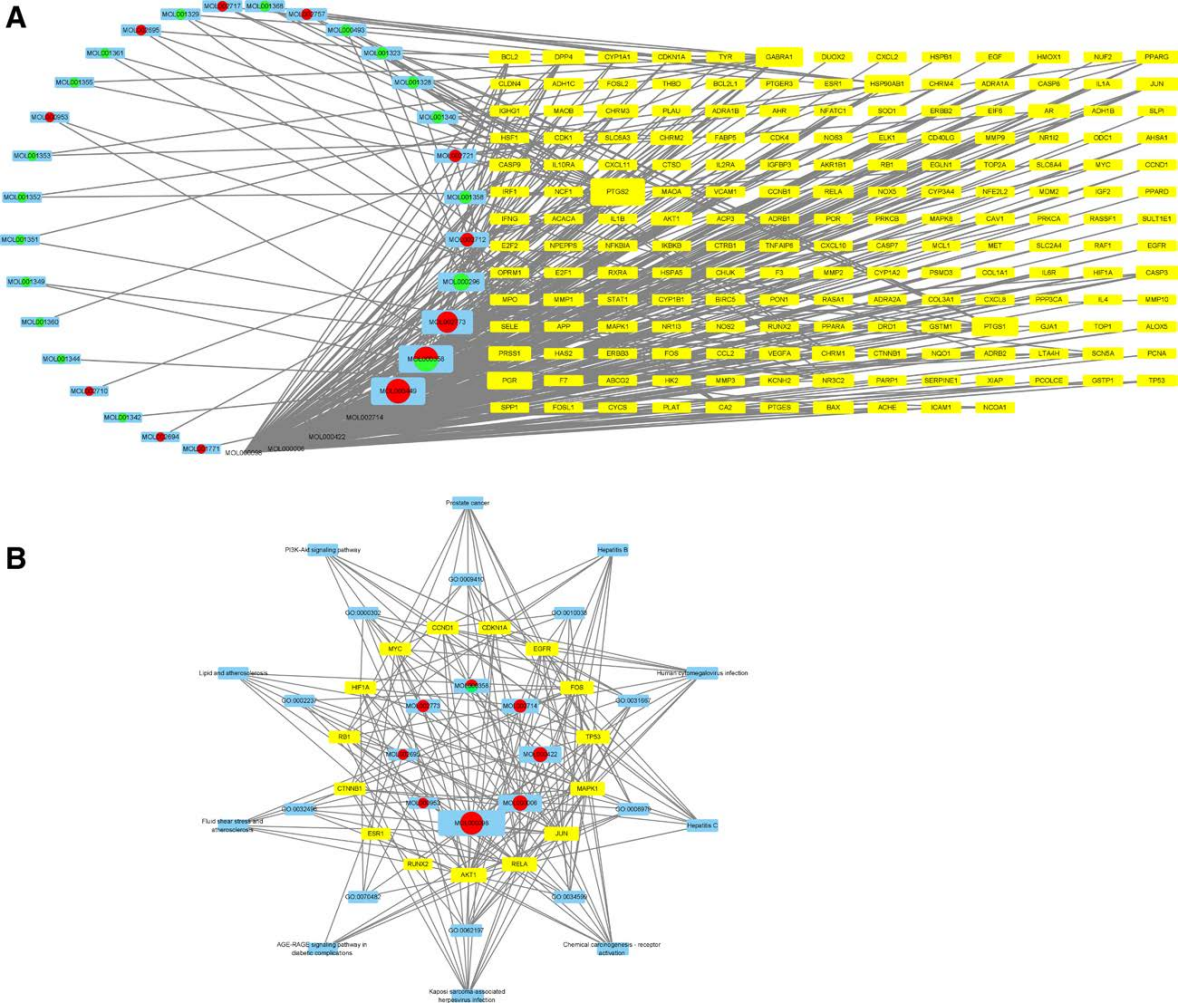
The construction of the network diagram. (A) Herbal-compounds-target network diagram. (B) Compounds-core targets-GO biological processes-signal pathways network diagram. GO = Gene Ontology.

Cytoscape 3.8.0 software was used for network construction. According to this network model, we conducted a scientific analysis of the relationships between the herbs, ingredients, targets, and diseases. We established a “herbal-compound-gene target” network diagram composed of 212 nodes and 415 edges. Through this diagram, we can easily observe the relationship between the herbs, ingredients, and targets, to reveal the potential pharmacological effects of PKSH. The multi-target and synergistic effects of the compatibility of PKSH can be observed through the network diagram (Fig. 2A).

### 3.4. PPI network construction and screening of core targets

Proteins related to diseases and PPI information can be found in the GeneBank and STRING databases 11.0. We input 154 target genes of PKSH for liver fibrosis into the STRING database and obtained a PPI network diagram of 154 nodes and 1212 edges with an interaction score ≥ 0.9. A total of 154 nodes represented the intersection genes between the target proteins of the active compound and disease-related genes, and 1212 edges represented interaction relationships.

To analyze the topological characteristics of PPI, we used the “analysis network” tool in Cytoscape to obtain the protein interaction network to obtain the relevant parameters based on the following 6 parameters: “Degree,” “Betweenness,” “Closeness,” “Eigenvector,” “LAC,” and “Network.” We selected the indexes above the median values as the key indices to obtain the core nodes of the PKSH. Finally, 15 hub nodes and 146 edges were obtained to establish the related network diagram. In order to investigate the relationship between components, core targets, GO biological processes (BPs), and KEGG signal pathways, we built a “Compounds-Core targets-GO biological processes-Signal Pathways” network diagram, which was composed of 8 compounds, 15 targets, 10 GO BPs, and 10 KEGG signal pathways (Fig. 2B).

**Figure 3.**
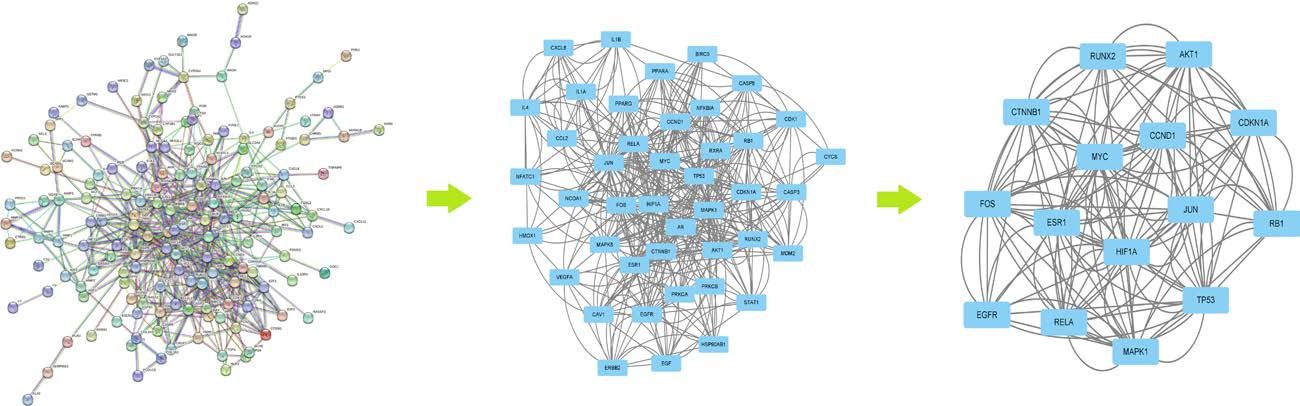
Map of protein interaction network and the identification of hub genes.

By analyzing the diagram, targets including EGFR, AKT1, MYC, RELA, JUN, MAPK1, FOS, HIF1A, CTNNB1, CCND1, ESR1, TP53, RUNX2, RB1, and CDKN1A may be core targets of the PKSH in the treatment of liver fibrosis (Fig. 3).

### 3.5. GO and KEGG enrichment analyses of core targets

After enrichment analysis of 179 hub targets, we got 2559 GO BPs and 168 KEGG pathways (*P* < .01). The GO database including BP, molecular function (MF), and cellular component (CC), was used to explore the potential biological molecular mechanisms.^[24]^ The KEGG database was used to identify biological functions and candidate targets.^[25]^

**Figure 4.**
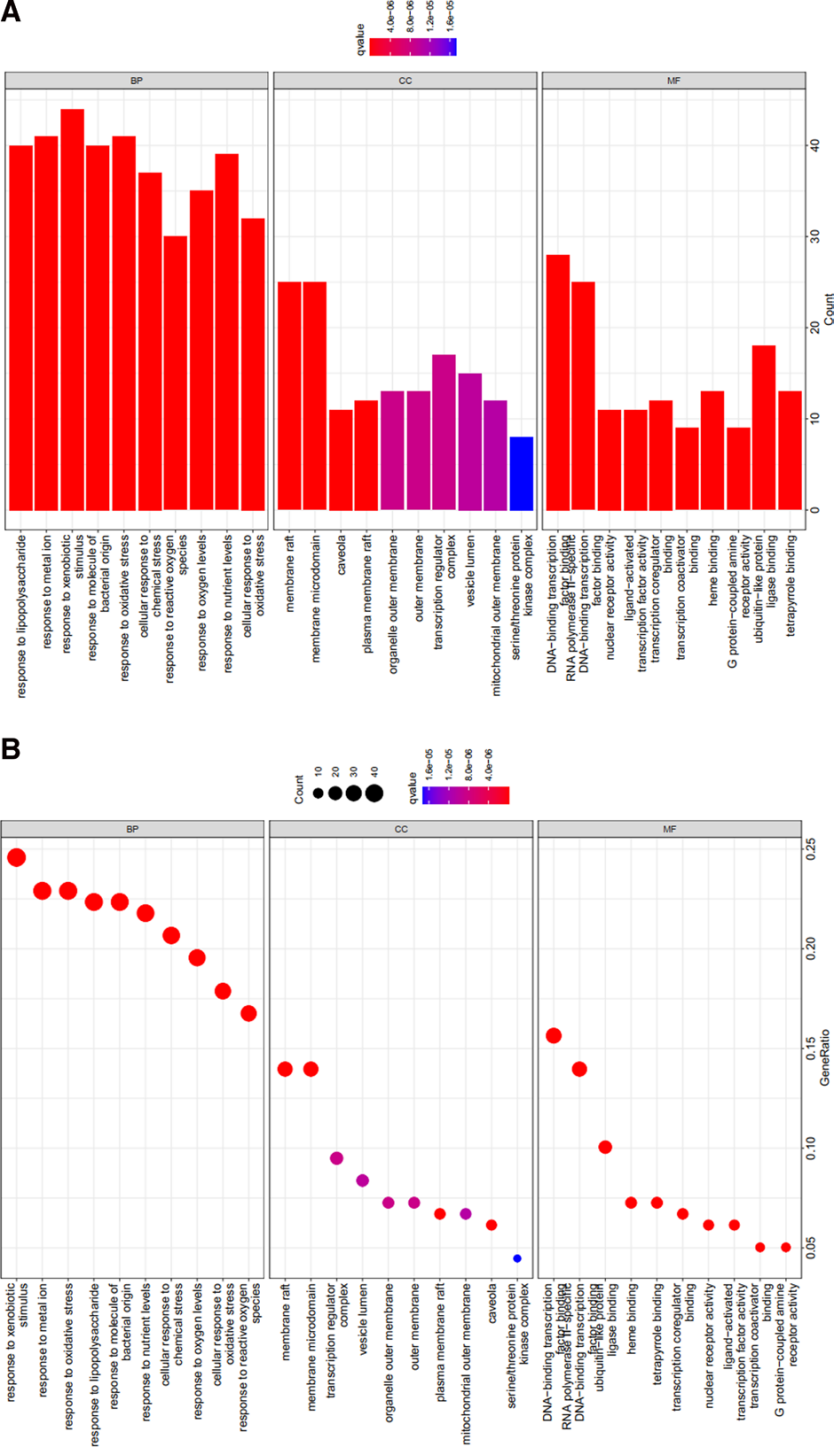
GO function analysis of the target proteins including BP, CC, and MF terms. (A) Bar chart. (B) Bubble chart. BP = biological process, CC = cellular component, GO = Gene Ontology, MF = molecular function.

According to the *P* value, 2559 GO BPs, including 2283 BP, 190 MF, and 86 CC terms, were found to be enriched in the GO enrichment analysis (*P* < .01). According to the *P* value, we selected the top 10 GO BPs for analysis, and the following were identified: (GO: 0009410) response to xenobiotic stimulus, (GO: 0010038) response to metal ion, (GO: 0006979) response to oxidative stress, (GO: 0032496) response to lipopolysaccharide, (GO: 0002237) response to molecule of bacterial origin, (GO: 0031667) response to nutrient levels, (GO: 0062197) cellular response to chemical stress, (GO: 0070482) response to oxygen levels, (GO: 0034599) cellular response to oxidative stress, and (GO: 0000302) response to reactive oxygen species (Fig. 4).

**Figure 5.**
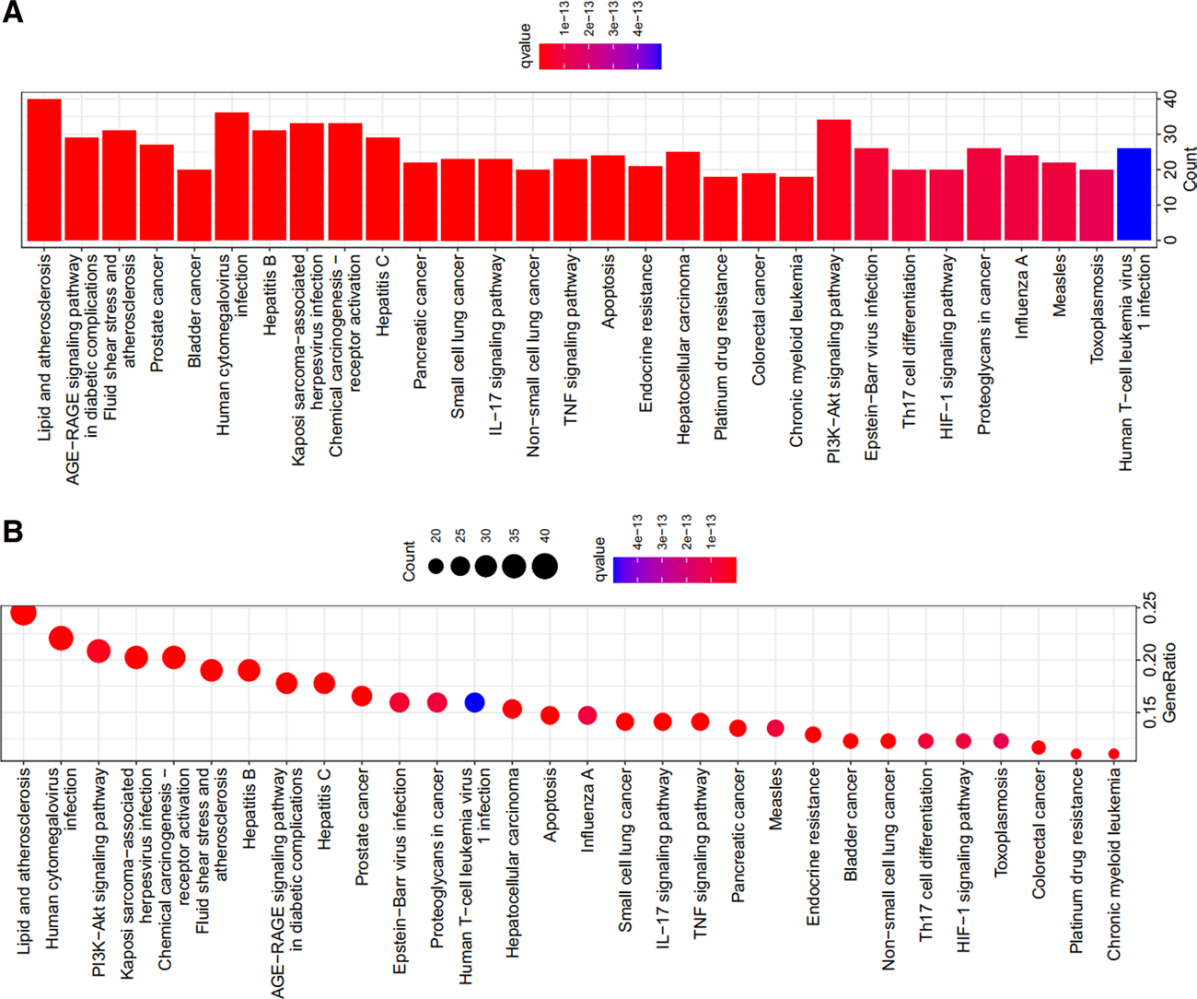
KEGG pathway enrichment analysis of the target proteins. (A) Bar chart. (B) Bubble chart. KEGG = Kyoto Encyclopedia of Genes and Genomes.

Furthermore, we selected the first 30 KEGG pathways for analysis, and the following KEGG pathways were identified: Lipid and atherosclerosis signaling pathway (hsa05417), human cytomegalovirus infection signaling pathway (hsa05163), PI3K-Akt signaling pathway (hsa04151), Kaposi sarcoma-associated herpesvirus infection signaling pathway (hsa05167), and chemical carcinogenesis receptor activation signaling pathway (hsa05207) (Fig. 5).

### 3.6. Docking simulation verification

**Figure 6.**
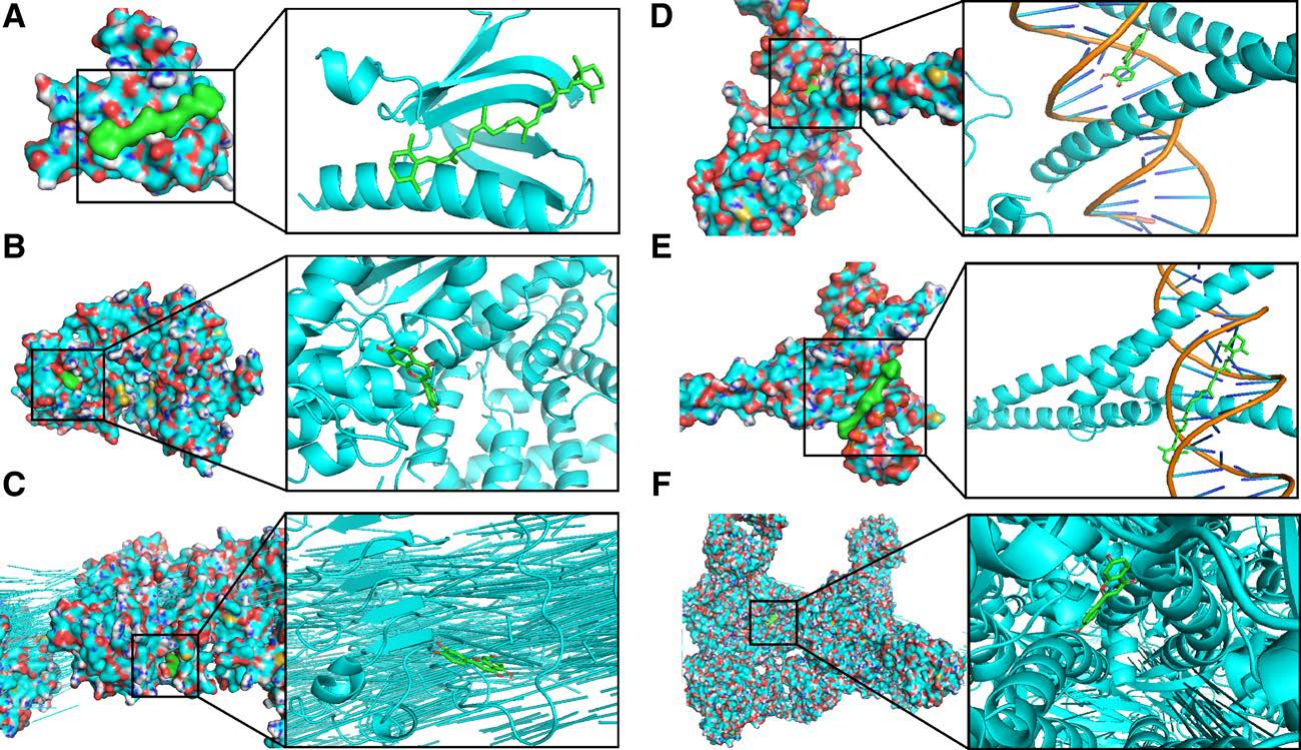
Molecular docking of the hub targets with bioactive compounds. (A) Binding poses of quercetin complexed with AKT1, affinity = −4.5 kcal/mol. (B) Binding poses of luteolin complexed with CCND1, affinity = −7.9 kcal/mol. (C) Binding poses of luteolin complexed with EGFR, affinity = −8.4 kcal/mol. (D) Binding poses of luteolin complexed with MAPK1, affinity = −9.1 kcal/mol. (E) Binding poses of quercetin complexed with MYC, affinity = −7.8 kcal/mol. (F) Binding poses of luteolin complexed with RELA, affinity = −8.4 kcal/mol.

AutoDockTools version 1.5.6 was used for molecular docking verification. Molecular docking analysis was used to validate the potential binding of the bioactive components in PKSH to disease-related targets. Because the PI3K-Akt signaling pathway was enriched in liver fibrosis disease in network pharmacology, we focused on the hub targets in the PI3K-Akt signaling pathway. Hub targets, including AKT1, CCND1, EGFR, MAPK1, MYC, and RELA, which were involved in the PI3K-Akt signaling pathway, were screened as intersecting targets and considered candidate genes for molecular docking. We used the main bioactive components, including quercetin and luteolin, to bind to the hub targets of the disease. The affinities of the combination of bioactive components and hub targets above are shown in Figure 6.

## 4. Discussion

The TCM PKSH is widely used to treat liver fibrosis in China; however, its mechanism of action remains unclear. In this study, we screened the core targets of PKSH and constructed a PPI network for the treatment of liver fibrosis. GO and KEGG enrichment analyses were performed to explore potential biological molecular mechanisms and identify biological functions and candidate targets. Through network pharmacological research, the potential pharmacological mechanism of PKSH in treating liver fibrosis was revealed, thereby providing a theoretical basis for subsequent drug development and pharmacological experiments. Through a network pharmacological study of the peach kernel and safflower in the treatment of liver fibrosis, we explored the potential pharmacological mechanism to provide a theoretical basis for subsequent drug research and development.

In this study, based on the results of network pharmacology, we speculated that the common ingredient β-sitosterol (MOL000358) in PKSH played a coordinating role in the treatment of liver fibrosis. Meanwhile, the main bioactive components, including quercetin and luteolin, could activate the PI3K-Akt signaling pathway by binding with the hub targets of disease and inhibiting liver fibrosis. By observing a Venny diagram of the disease and targets of PKSH, we identified 179 related genes and 15 hub genes. Overlapping genes between the target genes of active compounds and liver fibrosis-related genes, including PTGS2, GABRA1, PTGS1, HSP90AB1, PGR, CHRM1, PRSS1, CHRM2, CASP3, and DPP4, may play an important role in the treatment of liver fibrosis. Subsequently, 15 hub targets including EGFR, AKT1, MYC, RELA, JUN, MAPK1, FOS, HIF1A, CTNNB1, CCND1, ESR1, TP53, RUNX2, RB1, and CDKN1A in PPI network were obtained using Cytoscape software. Meanwhile, several typical targets and pathways in PKSH, including Lipid and atherosclerosis and PI3K-Akt signaling pathways, may contribute to the treatment of liver fibrosis. By constructing the Drug-Compound-Target-Diseases Network, we revealed that multiple ingredients in PKSH affected liver fibrosis-related targets and predicted potential signaling pathways in the treatment of liver fibrosis.

A total of 2559 GO BPs, including 2283 BP, 190 MF, and 86 CC terms, were found to be enriched in GO enrichment analysis. (GO: 0006979) response to oxidative stress in GO BPs is the main BP for the treatment of liver fibrosis. Highly expressed hub genes are associated with lipid and atherosclerosis and PI3K/Akt pathways to treat liver fibrosis.

Previous studies have indicated that PKSH contributes to pharmacological effects, including improving blood circulation, preventing atherosclerosis, regulating menstruation, and relieving pain.^[26]^ In the current study, we performed a network pharmacology analysis to explore the mechanism of PKSH in the prevention of liver fibrosis. Our results revealed that 33 key ingredients were screened and predicted as the active ingredients of PKSH. According to the degree of composition, the components of PKSH are quercetin (MOL000098), luteolin (MOL000006), kaempferol (MOL000422), baicalein (MOL002714), and stigmasterol (MOL000449). We used the main bioactive components, including quercetin and luteolin, to bind to the hub targets of the disease. The results revealed that quercetin and luteolin could activate the PI3K-Akt signaling pathway and inhibit liver fibrosis by binding to hub targets, including AKT1, CCND1, EGFR, MAPK1, MYC, and RELA. Quercetin has many functions, such as antioxidant, anti-inflammatory, and immune regulation, and many studies have confirmed the biological activity of quercetin in the treatment of liver fibrosis.^[27]^ Quercetin acts on 138 target genes related to liver fibrosis and plays an antifibrotic role in our PPI network; thus, it might be a promising therapeutic agent for liver fibrosis. Luteolin prevents the progression of liver fibrosis through multiple mechanisms.^[28]^ In our results, Luteolin acted on 53 target genes related to liver fibrosis and played an antifibrotic role in our PPI network. Kaempferol and baicalein have been proven to play antifibrotic roles.^[29]^ The common ingredient β-sitosterol (MOL000358) in PKSH also played a role in the treatment of liver fibrosis.

The formation mechanisms of liver fibrosis, which are caused by various factors, are complicated. The response to oxidative stress (GO: 0006979) in the BP played a vital role in the treatment of liver fibrosis. In the process of liver fibrosis, Kupffer cells recruit mononuclear cells, and hepatic stellate cells play a key role in liver fibrosis.^[30]^ Both the expression and synthesis of these inflammatory and pro-fibrogenic cytokines, which are secreted by these cells, are regulated mainly by oxidative stress. In addition, the participation of reactive oxygen species and lipid peroxidation products, such as the activation and action of stellate cells, can be clearly demonstrated in other basic events of liver fibrosis.^[31]^ Hence, the response to oxidative stress contributes to both the onset and progression of liver fibrosis, and PKSH may inhibit liver fibrosis by regulating oxidative stress reactions.

Research has indicated that the PI3K-AKT pathway is associated with the degree of liver fibrosis.^[32]^ Our results also revealed that the PI3K-Akt signaling pathway may be the main regulatory pathway for PKSH to play an inhibitory role in liver fibrosis. AKT and mTOR, key molecules of the PI3K pathway, can inhibit the activation of HSC and liver fibrosis. Therefore, PKSH may inhibit the activation of HSC by inhibiting the key molecules AKT and mTOR in the PI3K/AKT signaling pathway, thus inhibiting liver fibrosis.

Although network pharmacology has many advantages, it has some limitations. First, the reliability of network pharmacology needs to be further verified for network pharmacology is based on databases and computer analysis. Second, some active drug components identified by network pharmacology do not play important roles in clinical practice. Third, network pharmacology cannot detect the efficacy of drugs, which requires experimental verification.

In summary, we demonstrated that PKSH has a notable effect on inhibiting liver fibrosis. The main bioactive components in PKSH, including quercetin and luteolin, could activate the PI3K-Akt signaling pathway by binding with the hub targets of disease and inhibit liver fibrosis. The mechanism of action may regulate the response to oxidative stress through the PI3K-Akt signaling pathway, which may provide insights into drug development and follow-up pharmacologic research for liver fibrosis.

## Acknowledgments

We would like to acknowledge the support of the Department of No. 1 Surgery, The First Hospital Affiliated to Anhui Chinese Medical University, China.

## Author contributions

**Conceptualization:** Long Huang.

**Data curation:** Long Huang, Qingsheng Yu.

**Formal analysis:** Long Huang, Hui Peng.

**Funding acquisition:** Qingsheng Yu.

**Investigation:** Long Huang.

**Methodology:** Long Huang.

**Project administration:** Long Huang, Qingsheng Yu.

**Resources:** Long Huang, Hui Peng, Zhou Zhen.

**Software:** Long Huang, Hui Peng, Zhou Zhen.

**Validation:** Long Huang, Qingsheng Yu, Hui Peng, Zhou Zhen.

**Visualization:** Long Huang, Zhou Zhen.

**Writing – original draft:** Long Huang, Zhou Zhen.

**Writing – review & editing:** Long Huang, Zhou Zhen.
